# Supplementary material for: A Web-Based Automated Image Processing Research Platform for Cochlear Implantation-Related Studies
Source: J Clin Med. 2022 Nov 9;11(22):6640. doi: 10.3390/jcm11226640 (PMC9699139; doi:10.3390/jcm11226640)
Supplement: Supplementary file 1 [file jcm-11-06640-s001.zip › jcm-1982088-supplementary.pdf]

Supplementary information: A web-based automated image processing research platform for cochlear implantation related studies

Jan Margeta [1*], Raabid Hussain [2], Paula López Diez [3], Anika Morgenstern [4], Thomas Demarcy [2], Zihao Wang [5], Dan Gnansia [2], Octavio Martinez Manzanera [2], Clair Vandersteen [6], Hervé Delingette [5], Andreas Buechner [4], Thomas Lenarz [4], François Patou [2] and Nicolas Guevara [6,]

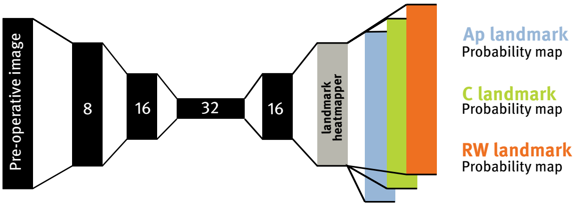

Figure S1. Landmark prediction model architecture. The architecture of our model presents a backbone built with 8, 16, 32 channels in the encoder and 16 channels in the decoder. The decoder is then directly connected to 3 output channels serving as landmark heatmaps that represent the probability maps for each landmark: C - the center of the basal turn of the cochlea , RW - the round window (defined at its center), and Ap - the apex (defined at the helicotrema). As input to the network we use 64x64x64 patches to train the network and similarly for inference in a sliding window fashion.

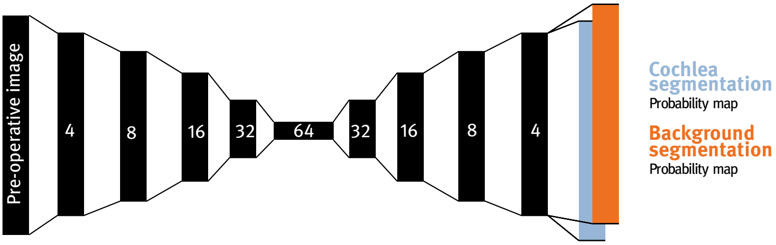

Figure S2. Pre-operative U-Net used for cochlear segmentation. The backbone is built with 4, 8, 16, 32, 64 channels in the encoder which we connect two convolutional heads: background prediction and cochlear structure prediction.

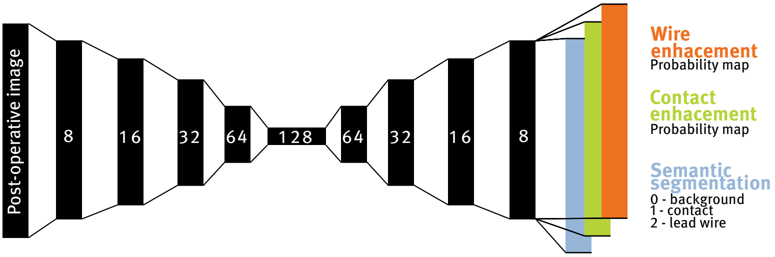

Figure S3. Post-operative U-Net for cochlear implant detection. The architecture of our model is shown in the figure where it can be seen that the backbone is built with 8, 16, 32, 64, 128 channels in the encoder. Onto the backbone which we connect three convolutional heads: semantic voxel segmentation task (background/contact/lead wire), contact enhancement task and array wire enhancement task.

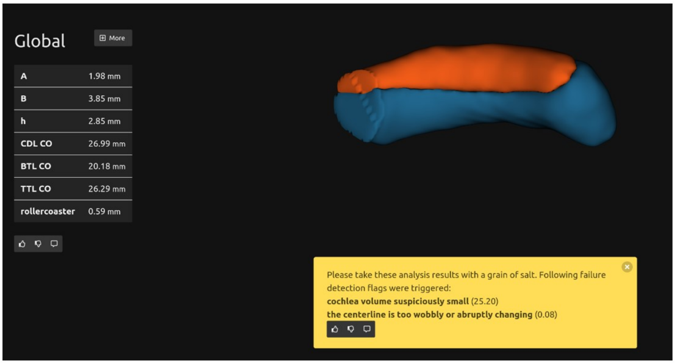

Figure S4. An example of a failure flag being triggered and shown to caution the user about possible processing failure. In this case a failure of the cochlear segmentation was detected.

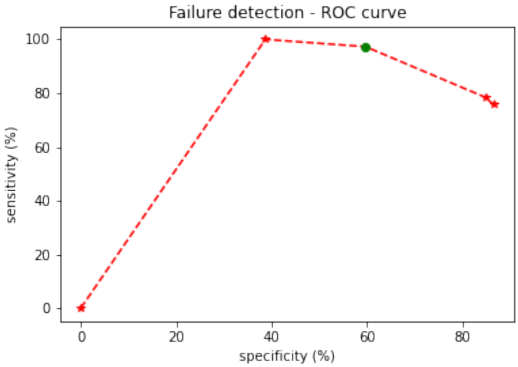

Figure S5. Receiver operating characteristic curve (ROC) curve for failure detection process. The threshold parameters of the configurations marked in green are currently implemented in the interface.

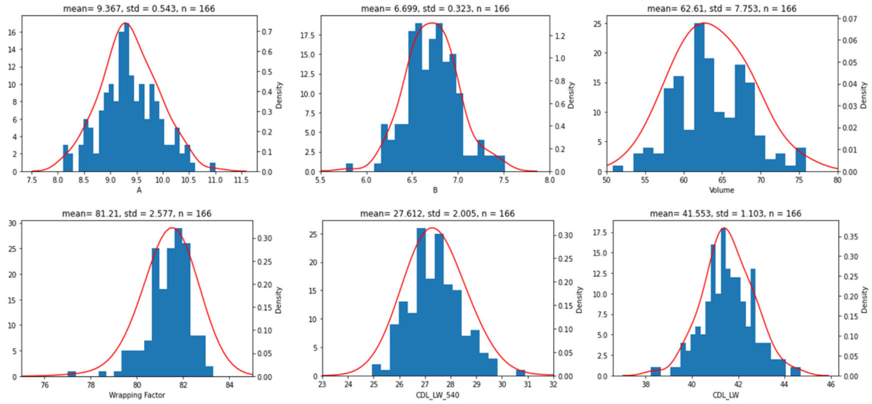

Figure S6. Pre-operative statistics from the qualitative assessment cohort automatically computed from the segmentations. The built-in data export allows further analysis.

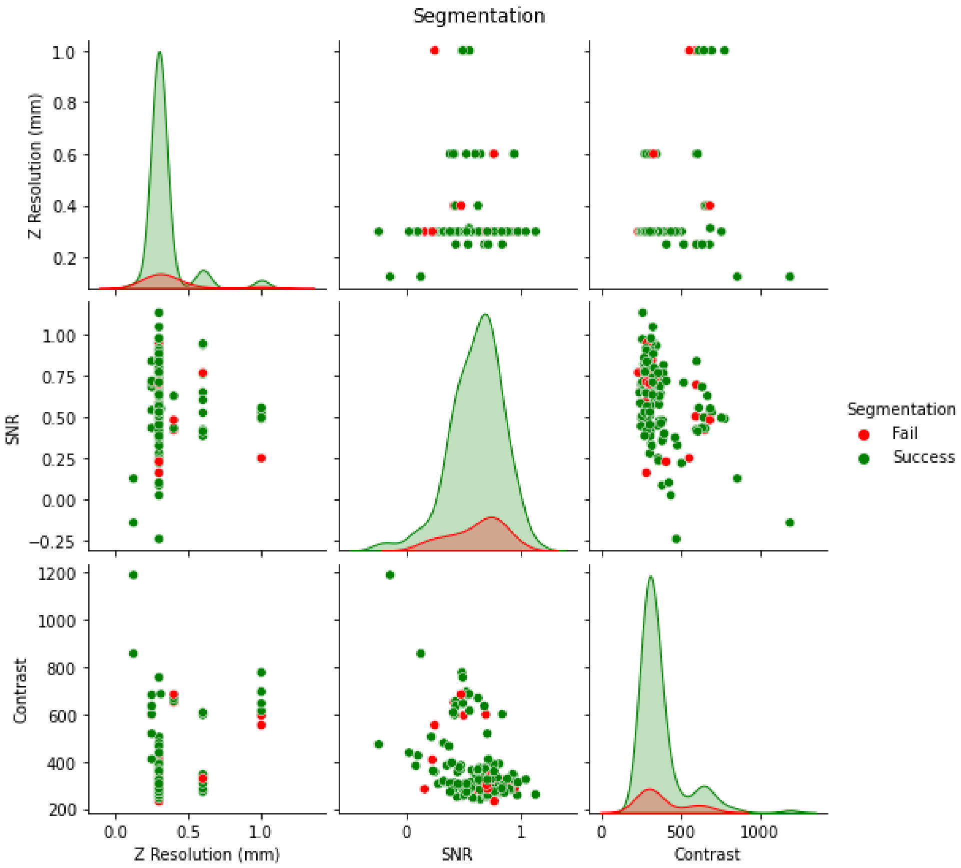

Figure S7. Qualitative segmentation performance (Reviewer 1) with respect to image quality criteria.

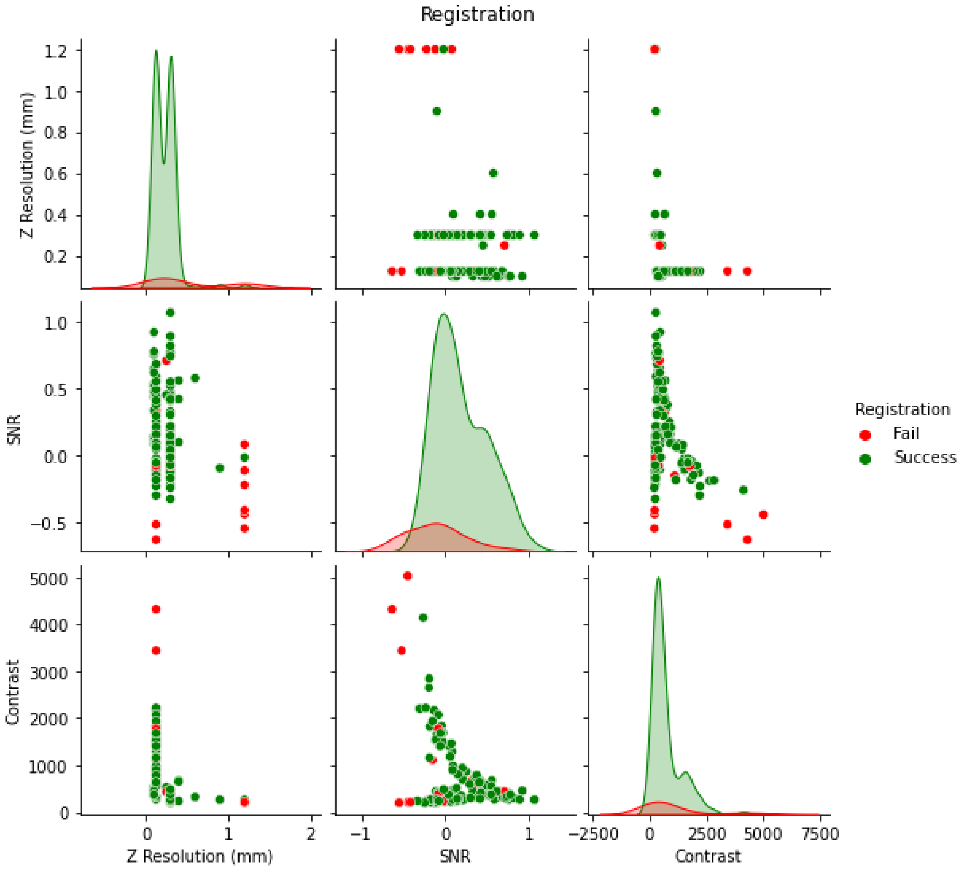

Figure S8. Qualitative registration performance (Reviewer 1) with respect to image quality criteria.

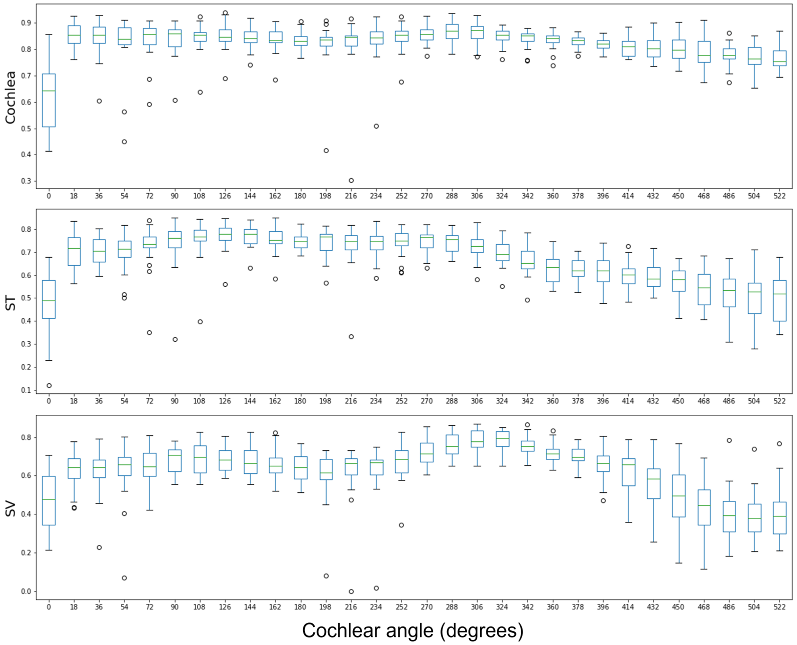

Figure S9. Dice scores per cochlear angle for the cadaver bone dataset (n=23). Our ST and SV segmentation pipeline achieves the best performance across the full basal turn (first 360°), with a decrease in performance towards the narrower and more ambiguous apical area.

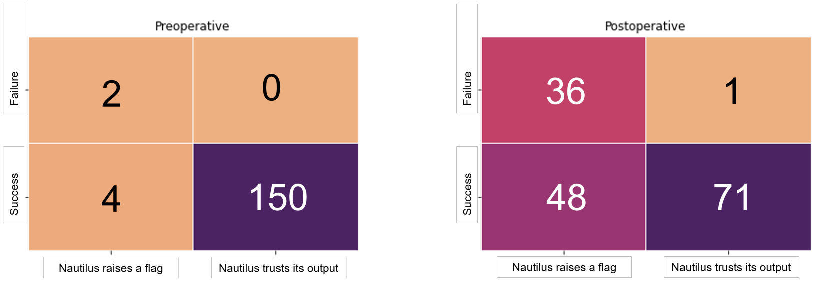

Figure S10. Quantitative evaluation of the failure detection pipeline with respect to reviewer 1's grading.
